# MicroRNA-3713 regulates bladder cell invasion via MMP9

**DOI:** 10.1038/srep32374

**Published:** 2016-08-31

**Authors:** Wen-Bo Wu, Wei Wang, Yi-Heng Du, Hao Li, Shu-Jie Xia, Hai-Tao Liu

**Affiliations:** 1 Department of Urology, Shanghai General Hospital, Shanghai Jiao Tong University School of Medicine, Shanghai 200080, China; 2 Department of Urology, Kashgar Prefecture Second People’s Hospital, Kashgar 844000, China

## Abstract

Transitional cell carcinoma (TCC) is the most common type of bladder cancer but its carcinogenesis remains not completely elucidated. Dysregulation of microRNAs (miRNAs) is well known to be involved in the development of various cancers, including TCC, whereas a role of miR-3713 in the pathogenesis of TCC has not been appreciated. Here, we reported that significantly higher levels of matrix metallopeptidase 9 (MMP9), and significantly lower levels of miR-3713 were detected in TCC tissue, compared to the adjacent non-tumor tissue, and were inversely correlated. Moreover, the low miR-3713 levels in TCC specimens were associated with poor survival of the patients. *In vitro*, overexpression of miR-3713 significantly decreased cell invasion, and depletion of miR-3713 increased cell invasion in TCC cells. The effects of miR-3713 on TCC cell growth appeared to result from its modification of MMP9 levels, in which miR-3713 was found to bind to the 3′-UTR of MMP9 mRNA to inhibit its protein translation in TCC cells. This study highlights miR-3713 as a previously unrecognized factor that controls TCC invasiveness, which may be important for developing innovative therapeutic targets for TCC treatment.

Transitional cell carcinoma (TCC) is the most common type of bladder cancer, in which the caner starts in the transitional epithelium of the bladder^1^. When the cancer grows into or through the other layers in the bladder wall, it becomes more advanced and difficult to treat. Over time, the cancer may grow outside the bladder and spread to nearby lymph nodes, or to other parts of the body as distal metastases, leading to poor prognosis^1^. This feature of TCC specifically emphasizes the importance of understanding the molecular mechanisms underlying the regulation of TCC invasiveness.

Epithelial-Mesenchymal Transition (EMT) is a critical biological event that triggers the modification of the cancer cell properties to allow cancer cell to adapt to a phenotype favoring invasiveness and metastases^2^,^3^,^4^,^5^. During EMT, cancer cells obtain capability of secreting proteinases from neighbor stromal cells or inflammatory cells for breaking through collagenous protein barriers. Matrix metallopeptidase 9 (MMP9) is an important matrix proteinase that degrades a basement membrane protein collagen type IV, and has been shown to be involved in the metastatic spread of various tumors, including TCC^6^,^7^,^8^,^9^,^10^,^11^. Nevertheless, the molecular pathway that regulates MMP9 activation has been shown to be different in different cancers. We recently reported that activation of WNT signaling pathway increased metastasis of TCC through activation of MMP9^12^. However, the signal pathways to control MMP9 activation in TCC has not been completely clarified.

MicroRNAs (miRNAs) are non-coding small RNAs that control some genes post-transcriptionally, through targeting the 3′-untranslated region (3′-UTR) of target mRNA^13^,^14^. Importantly, miRNAs have been found to control carcinogenesis and cancer progression^15^,^16^,^17^. Specifically, the miRNAs have been found to play a critical role in the tumorigenesis of TCC^18^,^19^,^20^,^21^,^22^,^23^,^24^,^25^, and in the control of MMP9 activation^26^,^27^,^28^,^29^,^30^,^31^. However, among all miRNAs, the function of miR-3713 has not been studied and previously reported.

Here, we examined the expression of MMP9 and miR-3713 in TCC tissues, and studied the association of miR-3713 levels with prognosis of the patients. We further showed the relationship between miR-3713 and MMP9 using bioinformatics analyses. We then overexpressed miR-3713 or inhibited miR-3713 in 2 commonly used TCC cell lines *in vitro* and studied their effects on MMP9 activation and TCC cell invasion.

## Materials and Methods

### Experimental protocol approval

All experimental protocols were approved by the Research Bureau of Shanghai General Hospital. All mouse experiments were approved by the Institutional Animal Care and Use Committee at Shanghai General Hospital (Animal Welfare Assurance). The methods regarding animals and human specimens were carried out in “accordance” with the approved guidelines. Informed consent was obtained from all subjects.

### Patient specimens

Surgical specimens from 28 TCC patients (all Stage IV) and matched adjacent non-tumor bladder tissues (NT) were obtained postoperatively in Shanghai General Hospital from 2011 to 2015. Informed consent was obtained from all subjects. All patients provided signed agreement for the resected tissue to be used for scientific research. The histology of the resected TCC specimens and control tissue were confirmed independently by senior pathologists. All patients were followed-up for 60 months.

### TCC Cell culture and transfection

Human TCC cell lines T24 and RT4 were both purchased from APCC (American Type Culture Collection, Manassas, VA, USA), and have been widely used in TCC research. T24 was generated from an 81-year-old female Caucasian^32^, and RT4 was generated from a 65-year-old male Caucasian^33^. Both cell lines were cultured in in RPMI1640 medium (Invitrogen, Carlsbad, CA, USA) supplemented with 15% fetal bovine serum (FBS; Sigma-Aldrich, St Louis, MO, USA) in a humidified chamber with 5% CO_2_ at 37 °C. MiRNAs mimics (miR-3713), miRNAs antisense oligonucleotides (as-miR-3713), null sequence, MMP9, and short hairpin small interfering RNA for MMP9 (shMMP9) were purchased from Origene (Beijing, China). The transfection was performed with 50 nmol/l plasmids, using Lipofectamine 2000 (Invitrogen). The transfection efficiency was more than 95%, based on expression of a GFP reporter.

### Transwell cell invasion assay

Cells (10^4^) were plated into the top side of polycarbonate transwell filter coated with Matrigel in the upper chamber of the BioCoatTM Invasion Chambers (Becton-Dickinson Biosciences, Bedford, MA, USA) and incubated at 37 °C for 22 hours. The cells inside the upper chamber with cotton swabs were then removed. Migratory and invasive cells on the lower membrane surface were fixed, stained with hematoxylin, and counted for 10 random 100X fields per well. Cell counts are expressed as the mean number of cells per field of view. Five independent experiments were performed and the data are presented as mean ± standard deviation (SD).

### MiRNA target prediction and 3′-UTR luciferase-reporter assay

MiRNAs targets were predicted with the algorithms TargetScan^34^. The data were analyzed as previously described^35^. The candidates were analyzed for context + score, which is the sum of the contribution of 6 features (including site-type contribution, 3′ pairing contribution, local AU contribution, position contribution, TA contribution and SPS contribution) (Supplementary Table 1). The MMP9 3′-UTR reporter plasmid (pRL-MMP9) and the MMP9 3′-UTR reporter plasmid with a mutant at miR-3713 binding site (pRL-MMP9-mut) were purchased from Creative Biogene (Shirley, NY, USA). TCC cells were collected 36 hours after transfection for dual-luciferase reporter assay (Promega, Fitchburg, WI, USA), according to the manufacturer’s instructions.

### Quantitative RT-PCR (RT-qPCR)

Total RNA was extracted from resected tissue specimens or from the cultured TCC cells, using miRNeasy mini kit (Qiagen, Hilden, Germany). Quantitative PCR (RT-qPCR) were performed in duplicates using QuantiTect SYBR Green PCR Kit (Qiagen), with the primers designed by Qiagen. A 2−△△Ct method was used to analyze and quantify the transcript levels. Values of gene transcripts were first normalized against housekeeping gene α-tubulin, and then compared to the experimental controls to gain relative expression values.

### Western blot

Western blot was performed as previously described^12^.

### Statistical analysis

The SPSS 18.0 statistical software package was used to analyze data in the current study. All values are depicted as mean ± standard deviation and are considered significant if p < 0.05. A one-way ANOVA method with a Bonferroni correction, followed by Fisher’ Exact Test, was applied. Kaplan-Meier analysis was used to analyze Patients’ survival.

## Results

### Association of miR-3713 levels in TCC specimens with prognosis of the patients

**Figure 1:**
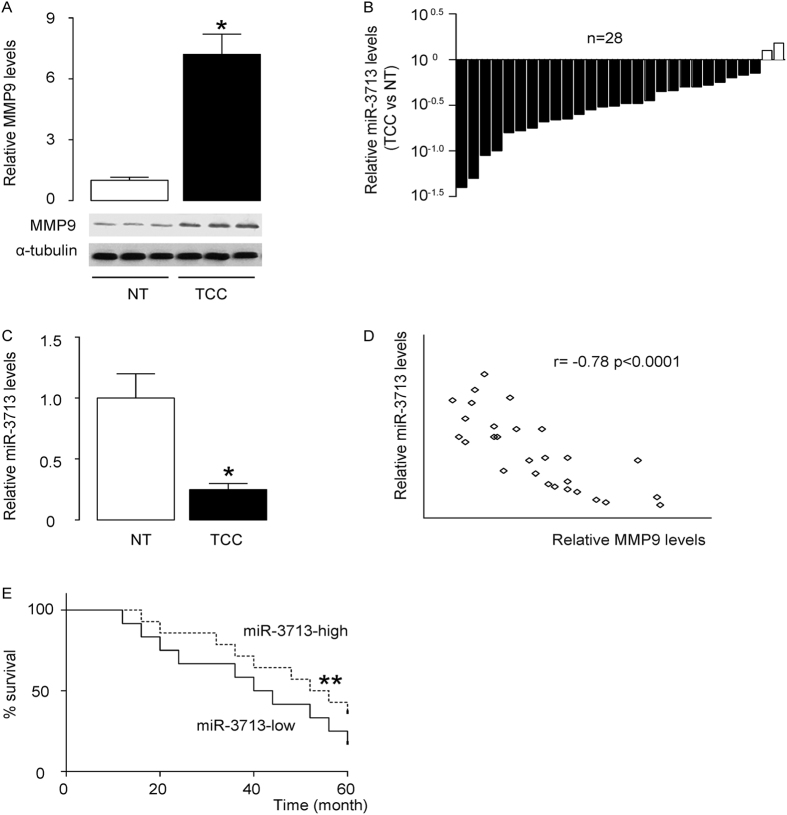
Low miR-3713 levels in TCC specimens associate with poor prognosis. (**A–C**) The levels of MMP9 and miR-3713 in 28 pairs of TCC tissues and adjacent non-tumor bladder tissues (NT) were measured by Western blot (**A**) and RT-qPCR, shown by individual values (**B**), and by mean ± SD (**C**). (**D**) A correlation test was performed between MMP9 and miR-3713, using the 28 TCC specimens. (**E**) The 28 TCC patients were followed-up for 60 months. The median value of all 28 cases was chosen as the cutoff point for separating miR-3713-high cases (n = 14) from miR-3713-low cases (n = 14). Kaplan-Meier curves were performed to compare 5-year survival between two groups. *p < 0.05. **p < 0.01. N = 28.

The levels of MMP9 and miR-3713 in 28 pairs of resected TCC tissues (Stage IV) and adjacent non-tumor bladder tissues (NT) were measured by Western blot and RT-qPCR, respectively. TCC specimens contained significantly higher levels of MMP9 (Fig. 1A), and significantly lower levels of miR-3713 (Fig. 1B,C). To test a possible relationship between miR-3713 and MMP9, we performed a correlation test in the 28 TCC specimens. A strong inverse correlation was detected (Fig. 1D, ɤ = −0.78, p < 0.0001, N = 28), suggesting a possible regulatory relationship between miR-3713 and MMP9 in TCC. In order to find out the clinical significance, these patients were followed-up for 60 months. The median value for miR-3713 in these patients was used as the cutoff point for separating miR-3713-high cases (n = 14) from miR-3713-low cases (n = 14). Kaplan-Meier curves were analyzed, showing that patients with low miR-3713 in TCC tissue had a significantly worse 5-year survival than those with high miR-3713 in TCC tisse (Fig. 1E). These data suggest that low miR-3713 levels in TCC specimens may associate with poor prognosis.

### MiR-3713 targets MMP9 to inhibit its protein translation in TCC cells

**Figure 2:**
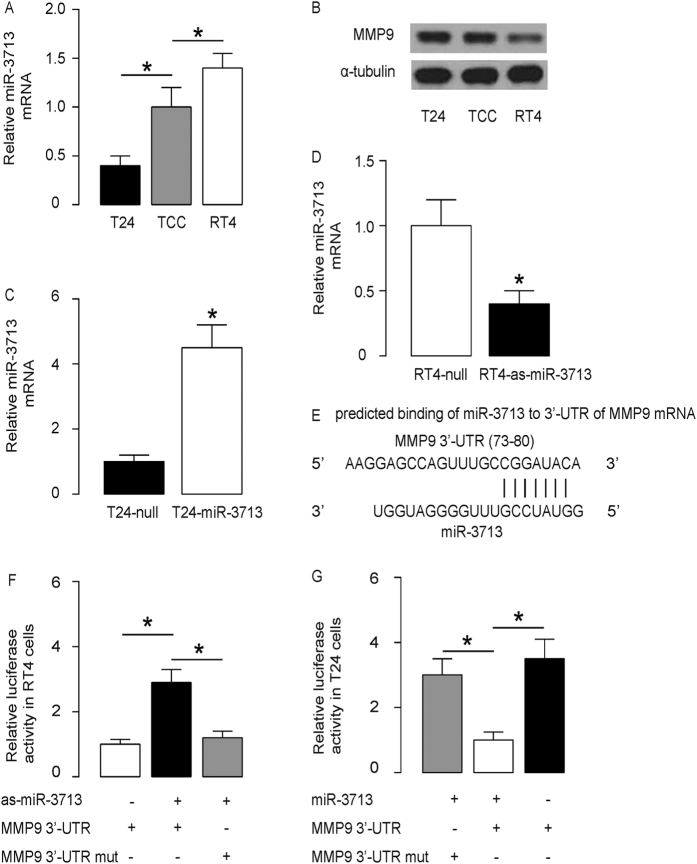
MiR-3713 targets MMP9 to inhibit its protein translation in TCC cells. (**A,B**) The levels of miR-3713 (**A**) by RT-qPCR and MMP9 (**B**) by Western blot in TCC cell lines RT4 and T24, compared to TCC tissue from patients. (**C**) T24 cells were transfected with miR-3713 mimics (miR-3713) or null as a control and examined for miR-3713 levels. (**D**) RT4 cells were transfected with antisense for miR-3713 (as-miR-3713) or null as a control and examined for miR-3713 levels. (**E**) Prediction of miR-3713-binding sites on MMP9 mRNA by bioinformatics algorithms. (**F–G**) The intact 3′-UTR of MMP9 mRNA (MMP9 3′-UTR), together with a 3′-UTR with mutant at miR-3713-binding site of MMP9 mRNA (MMP9 3′-UTR mut), was then cloned into luciferase reporter plasmids. Luciferase activity was determined in RT4 cells (**F**), which were co-transfected with 1 μg as-miR-3713/null plasmids and 1 μg MMP9 3′-UTR or MMP9 3′-UTR mut plasmids, and in T24 cells (**G**), which were co-transfected with 1 μg miR-3713/null plasmids and 1 μg MMP9 3′-UTR or MMP9 3′-UTR mut plasmids. *p < 0.05. N = 5.

Next, we examined miR-3713 and MMP9 levels in several TCC cell lines. Among these cell lines, we found that RT4 was a TCC cell line expressing relatively high miR-3713 and relatively low MMP9, while T24 was a TCC cell line expressing relatively low miR-3713 and relatively high MMP9 (Fig. 2A,B). Thus, we transfected T24 cells with miR-3713 mimics (miR-3713) (Fig. 2C), and transfected RT4 cells with antisense for miR-3713 (as-miR-3713) (Fig. 2D). The cells were also transfected with a null sequence as a control (null). The levels of miR-3713 in these modified TCC cells were assayed by RT-qPCR, 72 hours after transfection. The increases in miR-3713 levels in T24-miR-3713 cells (Fig. 2C) and the decreases in miR-3713 levels in RT4-as-miR-3713 cells (Fig. 2D) were confirmed. These miR-3713-modified TCC cells were used to examine the functional binding of miR-3713 to MMP9 mRNA as predicted by bioinformatics algorithms (Fig. 2E, Supplementary Table 1). The intact 3′-UTR of MMP9 mRNA (MMP9 3′-UTR), together with a 3′-UTR with mutant at miR-3713-binding site of MMP9 mRNA (MMP9 3′-UTR mut), was then cloned into luciferase reporter plasmids. First, RT4 cells were co-transfected with 1 μg as-miR-3713/null plasmids and 1 μg MMP9 3′-UTR or MMP9 3′-UTR mut plasmids (Fig. 2F). Next, T24 cells were co-transfected with 1 μg miR-3713/null plasmids and 1 μg MMP9 3′-UTR or MMP9 3′-UTR mut plasmids (Fig. 2G). The results demonstrate that miR-3713 specifically targets 3′-UTR of MMP9 mRNA to inhibit its translation in TCC cells.

### MiR-3713 decreases MMP9 protein but not mRNA in TCC cells

**Figure 3:**
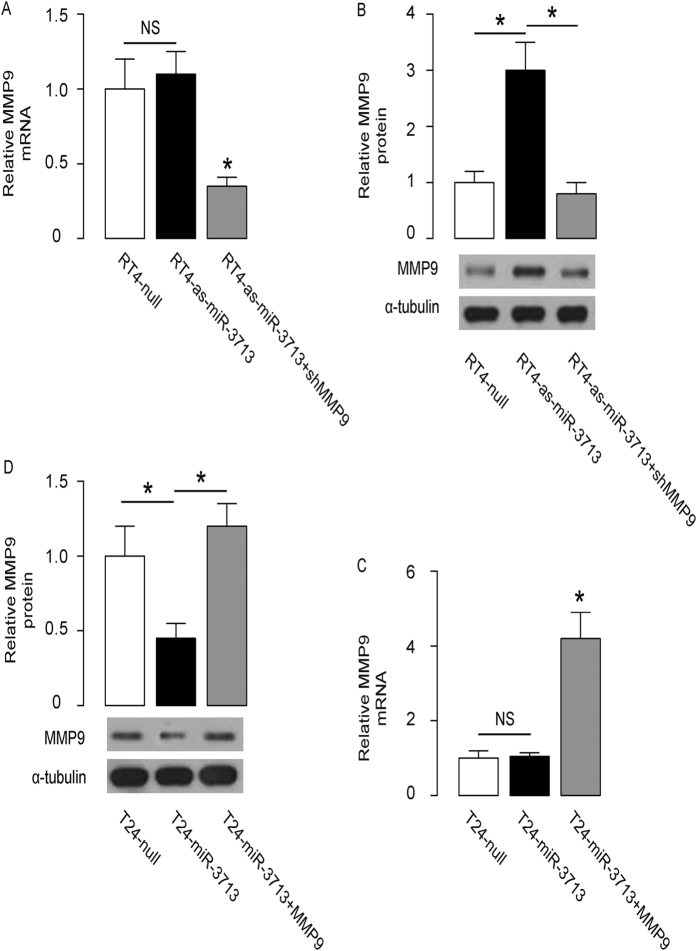
MiR-3713 decreases MMP9 protein but not mRNA in TCC cells. (**A,B**) The MMP9 levels in miR-3713-depleted (and MMP9-depleted) RT4 cells by RT-qPCR (A) and by Western blot (**B**). (**C,D**) The MMP9 levels in miR-3713-overexpressing (and MMP9-overexpressing) T24 cells by RT-qPCR (**C**) and by Western blot (**D**). *p < 0.05. NS: non-significant. N = 5.

The effects of miR-3713 on MMP9 were then analyzed in TCC cells. Although the MMP9 mRNA did not alter by miR-3713 depletion in RT4 cells (Fig. 3A), the MMP9 protein was significantly increased by miR-3713 depletion in RT4 cells (Fig. 3B). On the other hand, although the MMP9 mRNA did not alter by miR-3713 overexpression in T24 cells (Fig. 3C), the MMP9 protein was significantly decreased by miR-3713 overexpression in T24 cells (Fig. 3D). Together, these data suggest that miR-3713 may decrease MMP9 protein but not mRNA in TCC cells, consistent with abovementioned results from luciferase reporter assay.

### Modification of miR-3713 regulates TCC cell invasion

**Figure 4:**
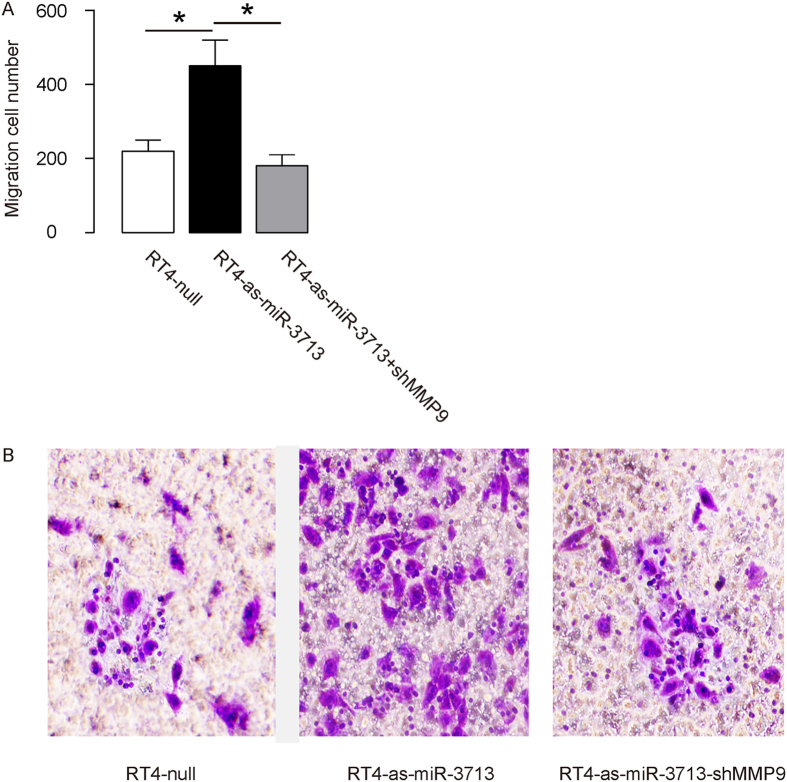
Depletion of miR-3713 abolishes RT4 cell invasion through MMP9. (**A–B**) RT4 cell invasion by miR-3713 depletion (and MMP9 depletion) in a transwell cell invasion assay, shown by quantification (**A**), and by representative images (**B**). *p < 0.05. N = 5.

**Figure 5:**
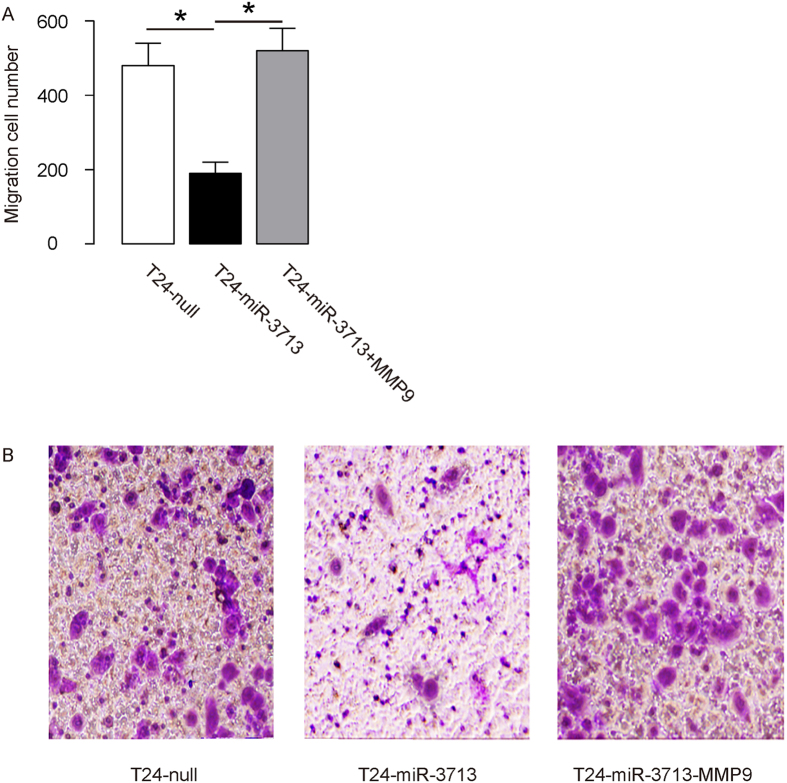
Overexpression of miR-3713 suppresses T24 cell invasion through MMP9. (**A–B**) T24 cell invasion by miR-3713 overexpression (and MMP9 overexpression) in a transwell cell invasion assay, shown by quantification (**A**), and by representative images (**B**). *p < 0.05. N = 5.

The effects of miR-3713 on the invasion of cultured TCC cells were then investigated. We found that miR-3713 depletion in RT4 cells significantly increased cell invasion in a transwell cell invasion assay (Fig. 4A,B). Similarly, miR-3713 overexpression in T24 cells significantly decreased cell invasion (Fig. 5A,B). Thus, modification of miR-3713 regulates TCC cell invasion.

### MiR-3713 regulates TCC cell invasion through MMP9

**Figure 6:**
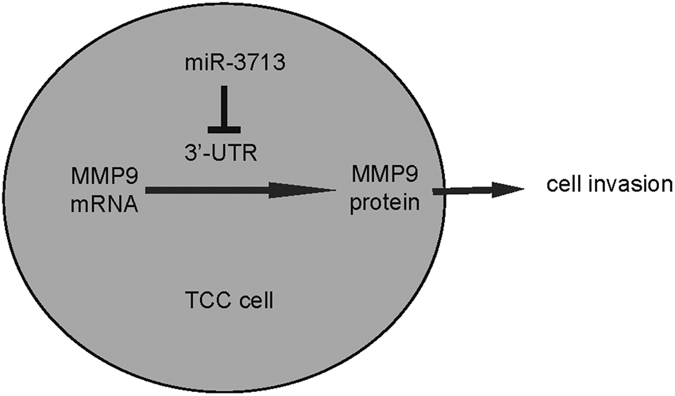
Schematic of the model. MiR-3713 inhibits TCC cell invasion, through translational suppression of MMP9.

In order to figure out whether miR-3713 may regulate TCC cell invasion through MMP9, we prepared plasmids for MMP9 overexpression (MMP9) and depletion (shMMP9). First, RT4-as-miR-3713 was further transfected with shMMP9, resulting in decreases in MMP9 mRNA (Fig. 3A) and protein (Fig. 3B) in these cells. Specifically, the effects of as-miR-3713 on MMP9 protein compromised the effects of shMMP9 on MMP9 protein, which explained the findings in TCC cells transfected with both as-miR-3713 and shMMP9. We found that MMP9 suppression abolished the effects of as-miR-3713 expression on cell invasion in RT4 cells (Fig. 4A,B). Next, T24-miR-3713 was further transfected with MMP9, which increased MMP9 mRNA (Fig. 3C) and protein (Fig. 3D) in these cells. Augmentation of MMP9 abolished the effects of miR-3713 expression on cell invasion in T24 cells (Fig. 5A,B). These data suggest that miR-3713 may regulate TCC cell invasion through MMP9 (Fig. 6).

## Discussion

MiRNAs play demonstrative roles in the carcinogenesis in various cancers. In line with these notions, their participation in the TCC progression has been widely reported^18^,^19^,^20^,^21^,^22^,^23^,^24^,^25^. For example, Jin *et al*. recently showed that overexpression of miR-192 significantly decreased the proliferation of bladder cancer cells. Moreover, miR-192-overexpressing cells exhibited a significant increase in G0/G1 phase and a significant decrease in S phase compared to the control miRNA-transfected cells. Furthermore, overexpression of miR-192 significantly induced apoptotic death in bladder cancer cells, increased the levels of p21, p27, and Bax, and decreased the levels of cyclin D1, Bcl-2, and Mcl-1^25^. In another study, Sun *et al*. showed that the levels of miR-138 were significantly decreased and the levels of ZEB2 were significantly increased in bladder cancer cells specimens, and were seemingly associated with cancer metastases. Moreover, suppression of miR-138 in bladder cancer cells may promote ZEB2-mediated cancer invasion and metastases^24^. These pioneering studies demonstrate microRNAs as an intriguing therapeutic target to prevent metastases of bladder cancer cells.

The role of MMP9 in TCC invasion and metastases has been well documented in the past studies^26^,^27^,^28^,^29^,^30^,^31^. However, the regulation of MMP9 by miRNAs in TCC has only been shown indirectly through other miRNA-targeting proteins, e.g. p53 by miR-221^36^, and c-met by miR-409-3p^37^. We actually screened all miRNAs that target MMP9 using bioinformatics analyses, and got 26 hits altogether. Among these candidates, we picked up the 4 that had the highest score and examined whether their expression levels may alter in TCC specimens compared to the normal tissue. We specifically found that miR-3713 was such a microRNA, as described in Supplementary Table 1. To the best of our knowledge, the current study is the first study that showed a direct regulation of MMP9 by a miRNA in TCC. Here, miR-3713 is a novel number of the miRNA family and has not been reported of any direct targets, including MMP9. In our previous report, we have shown that MMP9 is a key regulator of TCC invasiveness, and the WNT signaling pathway seems to regulate TCC metastasis through activation of MMP9^12^.

Here, we showed that MMP9 may be regulated by miR-3713 in TCC cells. Low level of miR-3713 in TCC tissues were associated with poor survival rate in TCC patients. Moreover, the levels of miR-3713 and MMP9 were inversely correlated. Then, we used a set of gain-of-function and loss-of-function experiments to show a regulatory relationship between miR-3713 and MMP9 in TCC cells. In the promoter luciferase assay that showed that bindings of miR-3713 to 3′-UTR of MMP9 mRNA inhibited protein translation, there seemed to be a significant repression of the mutant 3′-UTR in the presence of miR-3713. Given that the mutant 3′-UTR should not bind miR-3713 and yet did not have the same response as the wild type 3′-UTR plus antisense, these data may suggest the presence of at least one cryptic miR-3713 binding site. The large-scale changes in luciferase levels seemed unusual for miRNAs, which may be due to the importance of the binding site of miR-3713 on the 3′-UTR of MMP9 mRNA to its translation.

Besides regulation of MMP9 by miRNAs, MMP9 protein levels may be also affected by modulation of its degradation, e.g. through protein ubiquitination. Also, an exact role of WNT signaling in the molecular regulation of MMP9 by all these mechanisms may be studied In future to fully understand the control of MMP9 in TCC cell invasion. Future studies may also address the regulation of miR-3713 in TCC and confirm this model *in vivo*. Based on bioinformatics analyses, miR-3713 may have several interesting targets other than MMP9, e.g. mGAT4B, FOXK1, PFN2, ATXN1, which are promise new leads in the field of TCC.

To summarize, our study here may provide evidence for using miR-3713 as a novel target for treating TCC and contribute to the understanding of molecular regulation of MMP9-mediated TCC cell invasiveness.

## Additional Information

**How to cite this article**: Wu, W.-B. *et al*. MicroRNA-3713 regulates bladder cell invasion via MMP9. *Sci. Rep.* **6**, 32374; doi: 10.1038/srep32374 (2016).

## Supplementary Material

Supplementary Information
