# Supplementary material for: MicroRNA-3713 regulates bladder cell invasion via MMP9
Source: Sci Rep. 2016 Aug 31;6:32374. doi: 10.1038/srep32374 (PMC5006037; doi:10.1038/srep32374)
Supplement: Supplementary Information [file srep32374-s1.pdf]

MicroRNA-3713 regulates bladder cell invasion via MMP9

Wen-Bo Wu[1&], Wei Wang[1&], Yi-Heng Du[1], Hao Li[1], Shu-Jie Xia[1], Hai-Tao Liu[12*]

1 Department of Urology, Shanghai General Hospital, Shanghai Jiao Tong University School of Medicine, Shanghai 200080, China.

2 Department of Urology, Kashgar Prefecture Second People's Hospital, Kashgar 844000, China

& Wen-Bo Wu and Wei Wang contributed equally to this work.

* Correspondence: Hai-Tao Liu: 1 Department of Urology, Shanghai General Hospital, Shanghai Jiao Tong University, 100 Haining Rd, Shanghai 200080, China. 2 Department of Urology, Kashgar Prefecture Second People's Hospital, Kashgar 844000, China. Tel: +862163240090, Email: liuhaitao4@163.com

Run title: miR-3713 inhibits TCC via MMP9

Supplementary table 1: Prediction of human MMP9-binding miRNAs by Bioinformatics

predicted miRNAs	position at MMP9 3'-UTR	site-type contribution	3' pairing contribution	local AU contribution	position contribution	TA contribution	SPS contribution	context+ score
hsa-miR-3713	73-80bp	-0.247	0.003	0.055	-0.096	-0.083	-0.024	-0.39
hsa-miR-1224-3p	117-123bp	-0.12	-0.044	0.083	-0.052	0.023	-0.079	-0.19
hsa-miR-4690-3p	120-126bp	-0.12	-0.007	0.102	-0.052	0.016	-0.08	-0.21
hsa-miR-3123	179-186bp	-0.247	-0.018	0.021	-0.112	0.031	0.072	-0.25
hsa-miR-183	25-31bp	-0.074	0.001	0.034	-0.047	0.002	-0.014	-0.1
hsa-miR-4802-3p	26-32bp	-0.074	0.001	0.047	-0.047	0.007	0.03	-0.04
hsa-miR-4666-5p	43-49bp	-0.12	0.021	0.093	-0.056	-0.013	-0.034	-0.11
hsa-miR-133b	43-49bp	-0.12	0.021	0.093	-0.056	-0.013	-0.034	-0.11
hsa-miR-133a	47-53bp	-0.12	0.003	0.098	-0.055	-0.018	-0.028	-0.12
hsa-miR-296-3p	56-62bp	-0.074	-0.006	0.05	-0.044	0.026	-0.048	-0.1
hsa-miR-3667-3p	58-64bp	-0.12	-0.025	0.081	-0.054	0.003	-0.033	-0.15
hsa-miR-4448	61-67bp	-0.12	0.003	0.025	-0.053	0.016	-0.069	-0.15
hsa-miR-149	62-68bp	-0.12	-0.007	0.057	-0.053	-0.008	-0.047	-0.16
hsa-miR-892b	67-73bp	-0.12	0.012	0.033	-0.052	0.01	-0.016	-0.13

The site-type contribution reflects the average contribution of each site type. The 3' pairing contribution reflects consequential miRNA-target complementarity outside the seed region. The local AU content reflects the transcript AU content 30 nt upstream and downstream of predicted site. The position contribution reflects the distance to the nearest end of the annotated UTR of the target gene. The target site abundance contribution (TA) to context+ score reflects the abundance of target sites of a miRNA family in the set of distinct 3' UTRs. The seed-pairing stability contribution (SPS) to context+ score reflects the stability of a miRNA-target duplex, which is a function of the concentration of (A+U) in the seed region. The context+ score for a specific site is the sum of the contribution of 6 features [including site-type contribution, 3' pairing contribution, local AU contribution, position contribution, TA contribution and SPS contribution]. For each predicted target of each miRNA, the sum of the context+ scores for the sites to that miRNA was calculated as the total context+ score. Predicted targets of each miRNA family are sorted by total context+ score. The representative miRNA is the miRNA in its family with the most favorable (lowest) total context+ score. From the 4 candidates with the highest score (in blue), only miR-3713 was detected in human TCC specimens and paired non-tumor tissue. The other 3 microRNAs were not expressed.
